# Building bridges in migraine management: consensus pathways on best practices across primary and specialist care in Italy

**DOI:** 10.1007/s10072-026-09088-z

**Published:** 2026-05-19

**Authors:** Piero Barbanti, Cristina Tassorelli, Fabrizio Vernieri, Francesco De Cesaris, Roberto De Icco, Cherubino Di Lorenzo, Cinzia Finocchi, Licia Grazzi, Simona Guerzoni, Edoardo Mampreso, Tecla Mastronuzzi, Roberta Messina, Raffaele Ornello, Renata Rao, Daiana Taddeo, Marina De Tommaso, Innocenzo Rainero

**Affiliations:** 1 Headache and Pain Unit, IRCCS San Raffaele, Rome, Italy; 2 San Raffaele University, Rome, Italy; 3 Department of Brain and Behavioral Sciences, University of Pavia, Pavia, Italy; 4 Headache Science & Neurorehabilitation Unit, IRCCS Mondino Foundation, Pavia, Italy; 5 Headache and Neurosonology Unit, Fondazione Policlinico Campus Bio-Medico, Via Alvaro del Portillo 200, 00128 Rome, Italy; 6 Department of Medicine and Surgery, Università Campus Bio-Medico Di Roma, Rome, Italy; 7 Headache Center and Clinical Pharmacology Unit, Careggi University Hospital, Florence, Italy; 8 Department of Medico-Surgical Sciences and Biotechnologies, Sapienza University of Rome, Polo Pontino, Latina, Italy; 9 Division of Neurology, San Paolo Hospital ATS Liguria, Savona, Italy; 10 Neuroalgology Unit, Fondazione IRCCS Istituto Neurologico Carlo Besta, Milan, Italy; 11 Digital and Predictive Medicine, Pharmacology and Clinical Metabolic Toxicology, Headache Center and Drug Abuse, Laboratory of Clinical Pharmacology and Pharmacogenomics, AOU Policlinico Di Modena, Modena, Italy; 12 Headache Centre, Neurology - Euganea, Health Unit, Padua, Italy; 13 Italian College of General Practitioners and Primary Care (SIMG - Società Italiana Di Medicina Generale E Cure Primarie), Florence, Italy; 14 Neuroimaging Research Unit and Neurology Unit, IRCCS San Raffaele Scientific Institute, Milan, Italy; 15 Vita-Salute San Raffaele University, Milan, Italy; 16 Department of Biotechnological and Applied Clinical Sciences, University of L’Aquila, L’Aquila, Italy; 17 Department of Frailty and Continuity of Care, Headache Center-Neurology Unit, ASST Spedali Civili, Brescia, Italy; 18 Department of Clinical and Experimental Sciences, University of Brescia, Brescia, Italy; 19 DiBraiN Department, Neurophysiopathology Unit, University of Bari Aldo Moro, Bari, Italy; 20 Department of Neuroscience, Headache Center, University of Torino, Turin, Italy

**Keywords:** Migraine, General practitioner, Neurologist, Diagnosis, Management, Primary care

## Abstract

**Introduction:**

Migraine is a disabling neurological disorder often mismanaged, with only a minority of individuals receiving a timely correct diagnosis and appropriate treatment. The aim of this work was to establish consensus-based indications for improving the management of individuals with migraine and optimizing their journey from general practice to specialist care.

**Methods:**

A panel of 17 Italian experts, including neurologists, pharmacologists, and general practitioners (GPs) assessed a total of 36 statements addressing migraine diagnosis and management in primary care, referral pathways, and long-term care through the Delphi methodology.

**Results:**

The panel endorsed the role of GPs in the early identification and management of migraine, emphasizing the use of validated screening tools and headache diaries as well as the adoption of structured care pathways, where available, to ensure consistent and effective management. Specialist referral was emphasized for chronic migraine, medication overuse, or for individuals with inadequate response to preventive therapies. The panel advocated for a shared, long-term care model grounded in dynamic, bidirectional collaboration across healthcare levels to reduce the burden on tertiary headache centers.

**Conclusion:**

This consensus provides practical, context-specific guidance to facilitate care of migraine across primary and specialist care. Proper implementation of this model may streamline the management of individuals with migraine, reduce diagnostic delays, prevent unnecessary costs, and ultimately guide the development of a coordinated migraine care model in Italy.

## Introduction

Affecting over one billion individuals globally, migraine is among the leading causes of disability, and its burden has continued to grow over recent decades [1]. This trend underscores an urgent need to strengthen and better coordinate care strategies to address the complex clinical challenges posed by this condition. Migraine, characterized by recurring headache attacks accompanied by nausea, and/or photophobia, and phonophobia [2], imposes a substantial burden on individuals, society, and healthcare systems [3], and disproportionately affects women, reflecting the complex pathophysiological mechanisms involving hormonal, structural, and functional sex-related differences [4].

The International Classification of Headache Disorders (ICHD)-3 provides a reliable and validated framework for the diagnosis of migraine, distinguishing it from other primary and secondary headaches [2]. Once diagnosed, all individuals should receive adequate acute treatment for symptom relief, while preventive therapies should be prescribed following an individualized approach based on attack frequency, headache severity, response to acute medications, and overall disability [5–9].

With these premises, the diagnosis and initial treatment of migraine should take place within the primary care setting, following the principle that “the severity of illness determines the intensity of service” [10], thus allowing for a more efficient use of specialist resources [11–13]. General practitioners (GPs) are commonly the first healthcare professionals (HCPs) consulted by individuals presenting with headache and play a key role in the early assessment and longitudinal management of these patients [14].

Despite the availability of international and national recommendations to promote migraine diagnosis and care [6, 7, 11–13, 15, 16], real-world evidence indicates that many individuals either remain undiagnosed or experience diagnosis delays and suboptimal management [17]. In Italy, migraine burden is pronounced, with one of the highest age-standardized disability-adjusted life year (DALY) rates in Europe [1] and over one quarter of migraine individuals reporting severe disability [18]. Although many individuals turn to pharmacies for self-medication, approximately 50% of individuals with migraine do not consult any HCPs [19]. Among individuals with migraine consulting GPs, only a minority receive a correct diagnosis according to available data [17]; however, these estimates should be interpreted with caution, as they are influenced by heterogeneous study designs and do not fully account for factors such as delayed healthcare seeking, widespread self-medication, and variability in access to care across different settings. In addition, triptans and preventive therapies remain underutilized in real-world care [20, 21]. These observations underscore the need for expert-driven, pragmatic guidance to strengthen migraine care in Italian real-world practice. To this end, a panel of headache experts examined current barriers to timely migraine diagnosis and appropriate treatment and proposed practical system-oriented solutions through a structured Delphi process. The main objective was to provide guidance for the development of a coordinated migraine care model to facilitate migraine identification and management across levels of care.

## Methods

### The Delphi process

The Delphi technique is widely employed in clinical research to gain expert insights, address unmet needs, and develop consensus-based recommendations for clinical practice. Owing to the complexity and specificity of the topics discussed, Delphi studies involve specialists with demonstrated expertise in the subject area [22, 23]. A group of specialists, referred to as the steering committee, is responsible for formulating the statements, which are subsequently submitted for voting by a larger panel of experts.

The steering committee consisted of five Italian neurologists with established expertise in migraine (PB, MDT, IR, CT, FV). Twelve Italian physicians were invited as panelists; these comprised neurologists, a clinical pharmacologist affiliated with headache centers, and GPs. Panelists were selected based on their expertise in migraine management, while also ensuring geographical representation across Italy. The GPs involved in this Delphi process have specific expertise in neurological disorders, particularly migraine, and were intended to represent the broader network of Italian GPs. Consistent with previous reports in the literature [24], members of the steering committee also participated in voting on the statements, alongside the panel of experts. To ensure all participants were experts in migraine, two statements (20 and 32) were considered as internal controls to verify consistency and expertise.

### Statement development

During an online meeting held in February 2025, the steering committee discussed the key challenges and critical aspects to optimize migraine care across primary and specialist care settings. Drawing on current evidence and their clinical expertise, the members formulated 36 statements classified in four priority areas:

1. Diagnosis of migraine by GPs: 16 statements
2. Management of individuals with migraine within the primary care setting: 9 statements
3. Transition of care from primary to specialist services (including referral to a neurologist): 6 statements
4. Long-term, shared management of individuals with migraine between primary and specialist care: 5 statements.

Two methodologists assisted the steering committee through the Delphi process, without influencing the clinical content of the statements.

### Voting system, data collection, and analysis

The evaluation process was managed with the Butterfly Decisions AI-Assisted Decision-Making software platform (https://www.butterflydecisions.com). Each statement was presented to the panel through digital questionnaires based on a 4-point Likert scale (from “Completely disagree” to “Completely agree”). The choice of a 4-point Likert scale without a neutral option was intended to encourage panelists to express a clear position toward agreement or disagreement, thereby promoting a more decisive consensus process. Participants were also allowed to provide comments when relevant. The consensus threshold was predefined as a 70% agreement (or disagreement) among panelists. This threshold is in line with commonly used cut-offs in Delphi studies [25, 26]. The voting process was fully anonymous.

Data were analyzed using internal analytical tools and artificial intelligence agents integrated into the platform to perform semantic synthesis and clustering of textual feedback, to identify converging opinions, areas of disagreement, and potential reformulations of statements. The AI platform calculates proprietary metrics to measure the quality of the consensus such as certainty index, contradiction index, and sentiment analysis. The combination of these indices determines which statements require further revision. The output generated by the AI tools was subsequently verified by methodologists and the Steering Committee. The latter was fully responsible for interpreting the results and deciding on any modifications to the statements. This workflow ensured transparency, reproducibility, and methodological rigor in the consensus process [27, 28].

The first round of voting was conducted in May 2025; data were analyzed in June 2025. The steering committee then revised a selection of statements to explore the issues in greater depth and better delineate the extent of agreement. These revised statements were resubmitted for a second round of voting in September 2025, through the same online system. Final results were discussed with the extended voting panel in November 2025.

## Results

### Panelists

The voting panel was composed of five Italian migraine specialists from the steering committee (29.4%) and 12 additional Italian physicians (70.6%), including nine neurologists and one pharmacologist practicing in tertiary headache centers, and two GPs. Panelists were selected for their knowledge and expertise in migraine, which was confirmed by unanimous agreement to statements 20 and 32 (Table 1).

**Table 1:** Results from the first round of voting. Responses are expressed as percentages of agreement with each statement

Area 1: Diagnosis of migraine by the GP	
Statement	% agreement
1. Migraine is a common, debilitating, and treatable disorder; its understanding, identification, and management must be integral parts of GPs’ training and fall within their core competencies	94.1
2. During the initial consultation, the GP should collect the subject's medical history in a thorough and targeted manner, with a focus on:	
• Gender	100.0
• Age	100.0
• Family history of headache	100.0
• Number of headache days per month	100.0
• Use of symptomatic treatments	100.0
• Age at headache first onset	100.0
• Accompanying symptoms (e.g., nausea, vomiting, phonophobia, photophobia, osmophobia, worsening with physical activity)	100.0
• Presence of comorbidities	100.0
3. GPs have knowledge of migraine screening questionnaires	11.8
4. To promptly identify individuals with migraine during the initial consultation, the GP should use a rapid and validated screening tool, such as ID-Migraine [29]	88.2
5. In cases where chronic migraine is suspected, the GP may use the ID-Chronic Migraine [30] questionnaire as an alternative to ID-Migraine [29]	82.4
6. During the initial consultation, the GP should assess the individual's subjective pain intensity using the 11-point NRS	70.6
7. During the initial consultation, the GP should assess the quality of the pain as reported by the subject (e.g., pulsating, stabbing, pressing, constrictive, or other types)	100.0
8. During the initial consultation, the GP should apply the SNNOOP10 [37] criteria to exclude secondary causes of headache	94.1
9. The GP should encourage individuals to regularly complete a headache diary, documenting for each migraine episode:	
• The date	100.0
• The length	94.1
• The intensity	88.2
• The quality of pain	76.5
• The accompanying symptoms (nausea, vomiting, phonophobia, photophobia, osmophobia)	88.2
• Presence of aura	88.2
• Medications taken	100.0
10. Women of reproductive age should also record in the headache diary:	
• The days of menstrual bleeding and ovulation days (if known)	100.0
• The days on which hormonal contraceptives are taken (or applied)	94.1
11. The use of specific questionnaires (e.g., MIDAS) for assessing the level of disability is not mandatory in General Practice	76.5
12. The level of disability in individuals with headache can be assessed by the GP using the modified MIDAS questionnaire [31] (which includes three items related to days of school/work missed, social/family/leisure activities avoided, and days with reduced functioning to less than 50% due to migraine)	82.4
13. The GP should perform an appropriate clinical assessment of the individual with migraine, including, when necessary, blood tests if recent laboratory data are not available	94.1
14. The GP should measure blood pressure and can suggest that the subject record out-of-office blood pressure readings in the diary	100.0
15. The prescription of advanced diagnostic tests (e.g., neuroimaging) for migraine diagnosis should be avoided in General Practice	70.6
16. The GP should avoid prescribing unnecessary diagnostic tests and specialist referrals	100.0
Area 2: Management of individuals with migraine within the primary care setting	
17. The goals of primary care include:	
• The appropriate management of the acute phase	100.0
• The prevention of migraine progression (i.e., increase in the number of attacks per month)	94.1
18. The GP should refer to network plans or regional diagnostic-therapeutic pathways, when available, for the management of individuals with migraine	100.0
19. Lifestyle recommendations are an integral component of migraine management in General Practice and should include:	
• Sleep hygiene	100.0
• Regular physical activity	100.0
• Balanced diet	100.0
• Use of a headache diary to monitor migraine	100.0
• Stress reduction	94.1
20. Individuals with migraine experiencing fewer than four attack days per month, with mild to moderate intensity and disability, can be managed by the GP with acute treatment	100.0
21. Acute treatment is considered effective and should be continued if the subject, within 2 h of drug administration and for at least 24 h, reports all of the following conditions: 1) reduction of migraine intensity from moderate/severe to mild/absent in the majority of attacks (2 out of 3); 2) absence or minimal presence of associated non-painful symptoms; 3) absence of adverse events	100.0
22. The GP can prescribe preventive therapy if the subject meets at least one of the following criteria: 1) ≥ 4 days of disabling migraine per month; 2) inefficacy or contraindication to acute therapy; 3) excessive use of medications (≥ 10 doses of triptans or ≥ 15 doses of NSAIDs per month) for acute migraine treatment	94.1
23. It is appropriate to schedule the first follow-up visit:	
• Within 3 months of the initial consultation	64.7
• Between 3 and 6 months after the initial consultation	100.0
• More than 6 months after the initial consultation	47.1
24. In individuals with clinically stable migraine, in the context of primary care, scheduled follow-up is considered appropriate:	
• Every six months	52.9
• Tailored according to individual needs	100.0
25. At each follow-up visit, the GP should assess treatment use, effectiveness, and safety	100.0
Area 3: Transition from primary to secondary care: referral to specialist evaluation	
26. Chronic migraine (≥ 15 days per month for three months) requires specialist evaluation	100.0
27. An overuse of symptomatic medications (≥ 10 triptans or ≥ 15 NSAIDs per month) requires specialist evaluation	100.0
28. The ineffectiveness of oral preventive therapies, defined as a reduction of less than 50% in the number of monthly days with moderate to severe migraine, or as the absence of a clinically meaningful improvement as reported by the subject, requires specialist evaluation	100.0
29. The presence of relevant comorbidities (e.g., psychiatric or cardiovascular conditions) must be considered when deciding on referral to a specialist	88.2
30. The GP should refer the subject to a general neurologist as the initial level of specialist evaluation	82.4
31. Referral to a headache center is the responsibility of the general neurologist	70.6
Area 4: Long-term shared management between primary and secondary care	
32. It is suggested that a dynamic approach be adopted for the management of individuals with migraine, tailored to the clinical course of the condition and supported by bidirectional collaboration between levels of care, including referrals from secondary to primary care and vice versa	100.0
33. Each referral headache center should have a direct channel to provide support to GPs	88.2
34. The integrated use of the national EHR is recommended to facilitate communication and information sharing across different levels of care	100.0
35. The use of telemedicine is advised:	
• Within primary care	64.7
• Within community-based neurology care	70.6
• Within headache centers	88.2
36. It is suggested that artificial intelligence–based tools be implemented to optimize the management of individuals across different levels of care	94.1

*EHR* Electronic health record; *GP* General practitioner; *MIDAS* Migraine Disability Assessment; *NRS* Numerical Rating Scale; *NSAID* Nonsteroidal anti-inflammatory drug

### First round Delphi results

All statements reached formal consensus (> 70% agreement or disagreement), with four achieving consensus just above the predefined threshold (70.6%). Table 1 presents the distribution of responses obtained in the first round for each statement.

### Second round Delphi results

Following an evaluation of the first round of Delphi voting, the steering committee decided to rephrase three statements to clarify their content and refine the level of consensus. Table 2 shows the three rephrased statements and the corresponding second-round responses.

**Table 2:** Results from the second round of voting. Responses are expressed as percentages of agreement with each statement

Area 1: Identification of migraine by the GP	
Statement	Response
15. The prescription of complex diagnostic tests, particularly neuroradiological examinations, generally does not fall within the responsibilities of GPs, except in cases where there is a clinical suspicion of secondary headache, according to the SNNOOP10 [37] criteria, and in compliance with regional healthcare regulations and organizational frameworks	87.5% agreement
Area 3: Transition from primary to secondary care: referral to specialist evaluation	
31. Referral to a headache center falls within the responsibilities of the general neurologist, but it may also be carried out by the GP, depending on the local healthcare organization and the specific diagnostic–therapeutic care pathways in place	93.8% agreement
Area 4: Long-term shared management between primary and secondary care	
33. Does an efficient channel of communication currently exist between headache centers and GPs?	
Yes	6.2%
No	93.8%

*GP* General practitioner

### Area 1. Diagnosis of migraine by GPs

Panelists strongly agreed that migraine represents a common and debilitating condition; its recognition and management must form an essential part of GPs’ training and professional competencies. They also unanimously endorsed the need for collecting a comprehensive medical history at the first GP consultation for headache (statements 1–2, Table 1).

According to panelists, not all GPs may be aware of migraine screening questionnaires; nevertheless, the use of quick validated tools such as the ID-Migraine and, in cases of suspected chronic migraine, the ID-Chronic Migraine is suggested (statements 3–5, Table 1) [29, 30]. The use of the 11-point Numerical Rating Scale (NRS) for assessing pain intensity reached consensus just above the predefined threshold, while the assessment of pain quality was unanimously endorsed (statements 6–7, 70.6% and 100.0% agreement, respectively, Table 1). Panelists agreed that GPs should employ the SNNOOP10 criteria to screen for secondary headaches (statement 8, Table 1).

Diary keeping was considered essential. Subjects should be encouraged to maintain a headache diary documenting key features of attacks, including date, duration, pain intensity, quality, accompanying symptoms, aura, and medications taken, with agreement ranging from 76.5% to 100% across individual items (statement 9, Table 1). For women of reproductive age, menstrual cycle data and contraceptive use should also be recorded (statement 10, 100.0% and 94.1% agreement for the two options, respectively, Table 1).

Regarding the assessment of migraine-related disability, 76.5% of panelists agreed that the Migraine Disability Assessment (MIDAS) questionnaire should not be mandatory in general practice, whereas 82.4% agreed on the use of the modified MIDAS questionnaire [31] as a feasible tool for GPs (statements 11–12, Table 1).

Clinical examination and routine assessments in general practice are fundamental components of patient care; accordingly, appropriate blood tests and regular monitoring of blood pressure in individuals with migraine are suggested in general practice (statements 13–14, Table 1).

Consensus just above the predefined threshold was reached against prescribing advanced diagnostic tests in general practice (statement 15, 70.6% agreement, Table 1). In light of this result, the steering committee rephrased the statement to specify that, although the prescription of neuroradiological investigations should generally be avoided by GPs, such tests may be warranted in selected cases (e.g., when secondary headache is suspected). The revised statement achieved a greater consensus (statement 15, 87.5%, Table 2).

Avoiding unnecessary tests and referrals achieved full consensus after the first round of voting (statement 16, Table 1).

### Area 2. Management of individuals with migraine within the primary care setting

Consensus was achieved on the core goals of primary care for migraine management, these being: 1) appropriate management of the acute phase and 2) prevention of migraine progression (statement 17, 100.0% and 94.1% agreement for the two items, respectively, Table 1). Panelists unanimously agreed that GPs should follow structured care protocols when available (statement 18, Table 1).

Lifestyle interventions were considered integral components to migraine management in general practice. Consensus was reached regarding indications for sleep hygiene, regular physical activity, balanced diet, use of a headache diary (all 100.0% agreement), and stress reduction (94.2% agreement) (statement 19, Table 1).

Panelists agreed that therapeutic strategies should be tailored according to migraine frequency and severity. GPs should manage individuals experiencing fewer than four migraine days per month, with mild to moderate intensity and disability, with acute treatment (statement 20, Table 1). Acute treatment should be considered as clinically effective if subjects report, within two hours of administration and for at least 24 h, a meaningful reduction of pain intensity (moderate/severe to mild/absent in ≥ 2 out of 3 attacks), minimal or absent non-painful associated symptoms, and no adverse events (statement 21, Table 1). Panelists endorsed the use of preventive therapy for migraine individuals meeting at least one of the following criteria: i) ≥ 4 days of disabling migraine per month (episodes perceived as having a significant impact on daily life), ii) inefficacy or contraindication to acute therapy, iii) excessive use of acute medications (≥ 10 doses of triptans or ≥ 15 doses of nonsteroidal anti-inflammatory drugs [NSAIDs] per month) (statement 22, 94.1% agreement, Table 1).

The ideal timing of the follow-up after the initial GP consultation was deemed to be 3–6 months (statement 23, Table 1). For individuals with well-controlled and clinically stable disease, tailored follow-up based on individual needs was considered unanimously adequate (statement 24, Table 1). At each follow-up, the GP must assess treatment use, effectiveness, and safety (statement 25, Table 1).

### Area 3. Transition of care from primary to specialist services, including referral to a neurologist

Panelists agreed on specific clinical situations that should prompt GP referral to specialist care. These include chronic migraine (≥ 15 days per month for three months), excessive use of symptomatic medications (≥ 10 doses of triptans or ≥ 15 doses of NSAIDs per month), and ineffectiveness of traditional oral preventive therapies, defined as either a reduction of less than 50% in the number of monthly migraine days with moderate-to-severe headache or the absence of clinically meaningful self-reported improvement (statements 26–28, Table 1). Beyond these situations, panelists agreed that relevant comorbidities (e.g., psychiatric or cardiovascular conditions) should also be considered when deciding on referral to a specialist (statement 29, Table 1).

According to panelists, the most appropriate care pathway begins with GP referral to a general neurologist; onward referral to a headache center should be suggested by the neurologist (statements 30–31, Table 1). The latter statement reached a consensus just above the predefined threshold agreement and was modified to clarify that the GP may also directly refer migraine individuals to headache centers, where allowed by regional healthcare regulations and care pathways. In the second round of voting, the consensus reached 93.8% (statement 31, Table 2).

### Area 4. Long-term, shared management of individuals with migraine between primary and specialist care

Migraine management should adopt a dynamic approach, guided by the clinical course of the condition and supported by bidirectional collaboration between different levels of care (statement 32, Table 1). To strengthen collaboration between primary and specialist care, the use of direct communication channels between referral headache centers and GPs to provide ongoing support is suggested (statement 33, 88.2% agreement, Table 1). To further elaborate on this topic, the statement was revised to ask about the actual existence of such channels. The results highlighted a major gap, as only 6.2% reported the existence of direct communication systems between headache centers and GPs (statement 33, Table 2).

Full consensus was achieved on the integrated use of the national electronic health record (EHR) (Fascicolo Sanitario Elettronico) to enhance information sharing across all care levels (statement 34, Table 1). The role of telemedicine was supported in community-based neurology services and headache centers (statement 35, 70.6% and 88.2%, respectively, Table 1). Overall, the panel endorsed the implementation of artificial intelligence (AI)-based tools to optimize migraine management across levels of care (statement 36, Table 1).

## Discussion

**Fig. 1:**
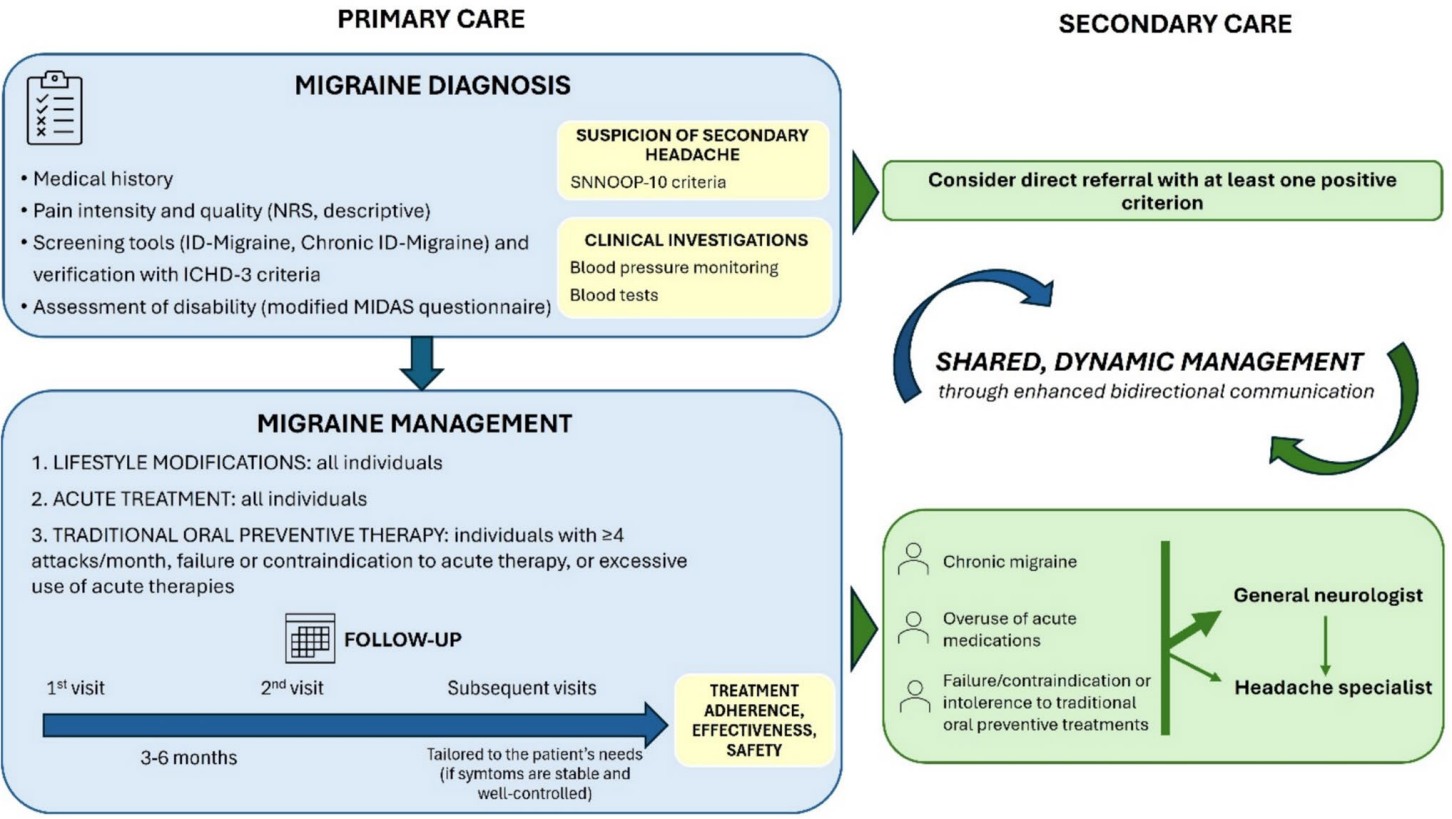
Towards optimal migraine care in the Italian healthcare system. A consensus roadmap across care levels. Workflow for the optimal identification and management of individuals with migraine in primary care and across higher care levels extrapolated by the statements. ICHD-3: International Classification of Headache Disorders-3; MIDAS: Migraine Disability Assessment; NRS: Numerical Rating Scale

Using the Delphi method this work reinforces the central role of GPs in the early diagnosis and longitudinal management of migraine and addresses key system-level barriers to optimal management across primary and specialist settings in Italy. The findings outline an ideal model of care that enables earlier diagnosis, delineates clearer referral pathways, and supports long-term, shared management between primary care and specialist services (Fig. 1). The implementation of this pathway may be challenging in routine primary care, as extended follow-ups and detailed monitoring may require additional time and organizational resources. Given time constraints, competing priorities, and variability in experience and resource availability among GPs, full implementation may not always be feasible. The proposed pathway represents an ideal framework of care rather than a prescriptive model. Its implementation in routine primary care should be adapted pragmatically to account for time constraints, competing priorities, and variability in organizational and clinical resources.

This coordinated approach is consistent with integrated care models that are being increasingly adopted for the management of other chronic diseases, such as type 2 diabetes and chronic respiratory conditions. Overall, these models aim to reduce fragmentation of care, with bidirectional flows across care levels according to disease complexity and severity to ensure continuity of care, supporting long-term management [32–34].

### The role of GPs in migraine identification

A satisfactory first consultation with the GP is crucial to ensure effective and continuous care [35]. In line with previous studies [6, 7, 11–13, 36], the key role of GPs in identifying migraine remains undisputed. During the initial consultation, GPs should proactively obtain a detailed medical history [2, 7, 11] to gain an exhaustive overview of the clinical profile and distinguish migraine from other headache disorders.

The panel strongly endorsed the use of ID-Migraine or ID-Chronic Migraine for screening purposes, as tools intended to complement the application of the ICHD-3 diagnostic criteria. Both are simple, rapid, and reliable tools that facilitate the identification of most individuals with migraine or chronic migraine, respectively [29, 30]. Hence, their application within primary care is considered valuable [7, 11]. Currently, there are no definitive instrumental diagnostic tests for migraine, further underscoring the importance of careful clinical evaluation [13]. Notably, panelists stressed the need for greater awareness and dissemination of these screening tools.

During the initial GP consultation, individuals with migraine should describe both the quality and intensity of their symptoms. While panelists supported using the NRS to assess pain intensity and promote consistent evaluation in primary care, some uncertainty remained about its implementation. In the absence of NRS use, individuals with migraine should document pain intensity and other relevant details descriptively in a headache diary. The headache diary is widely regarded as a relevant aid in confirming headache severity, monitoring headache frequency, identifying medication overuse, and guiding therapeutic decisions [7, 13, 15]. As such, GPs should encourage consistent daily use of the diary, which should remain simple and easy to interpret to maximize compliance [7]. Given the established association between migraine and fluctuations in female hormone levels [4], women should track additional data related to the menstrual cycle to better understand their connection with migraine episodes.

The assessment of migraine disability using the MIDAS questionnaire was deemed not mandatory in primary care. The modified three-item MIDAS was considered a more applicable tool for GPs to track changes in migraine-associated disability over time and understand medication effectiveness during follow-ups [7, 11, 12].

The role of GPs is critical in detecting suspected secondary headache disorders [7, 11–13, 15]. Employing the SNNOOP10 list during the initial consultation facilitates the identification of potential red flags. Although shorter alternatives exist (e.g., SNOOP4), the systematic application of the SNNOOP10 checklist in subjects presenting with a new headache disorder is expected to maximize the detection of potential secondary causes [37].

The suspicion of secondary causes of headache justifies the prescription of advanced diagnostic tests [6, 7, 11, 13], although these techniques should not be used for migraine diagnosis [7, 13], as they have been proven largely unnecessary [38]. Despite such recommendations, neuroimaging and other high-cost procedures continue to be overutilized in real-world care [17, 38]. In light of this evidence, the panel discouraged the routine use of such tests for diagnostic purposes and, more broadly, the prescription of unnecessary investigations and referrals, except in cases where a secondary headache is suspected. Instead, a thorough clinical assessment was emphasized, including blood test analyses to help exclude conditions associated with headache, such as secondary headache disorders or migraine-associated comorbidities (i.e., thyroid dysfunction [39] and immunological disorders [40]). In addition, GPs should measure blood pressure, and individuals with migraine should be encouraged to record out-of-office blood pressure values in their diary [15]. Although the relationship between hypertension and migraine is not yet fully understood, hypertension is thought to contribute to the progression towards chronic migraine, and antihypertensive drugs are among the recommended migraine preventive therapies [7, 11, 13].

### The role of GPs in migraine management

In most cases, the initial management of migraine should be addressed in primary care, thereby contributing to appropriate access to higher-level healthcare services [13]. Once the diagnosis of migraine has been formulated, the GP should address acute attack–related symptoms and consider preventive therapies to reduce disease burden and prevent progression. Given the decentralized organization of the Italian healthcare system, panelists emphasized the importance of following regional diagnostic-therapeutic pathways or locally defined care plans, where available. Although such pathways have been successfully developed in some Italian regions [41], their structure remains mostly heterogeneous and their implementation in routine clinical practice is still limited [24]. In this context, GP adherence to structured care protocols is key to enhancing the appropriate management of individuals with migraine and streamlining referral processes.

Lifestyle recommendations are the cornerstone of migraine management in general practice and should be equally applied, as these measures can have a broader beneficial impact on health [42]. These non-pharmacological interventions can empower individuals with migraine in the self-management of their condition; as such, they should be thoroughly discussed during the initial consultation and follow-up visits.

Individuals with migraine should be primarily managed with analgesics or NSAIDs and triptans in primary care [8, 16]. In cases of insufficient or inadequate response, contraindication, or overuse of acute treatments, preventive medications should be started [9]. Preventive options include antidepressants, anticonvulsants, antihypertensives, beta-blockers, and calcium channel blockers, drug classes commonly prescribed in primary care for conditions other than migraine. Italian regulations require the failure of at least three preventive treatments to access reimbursement of anti-calcitonin gene-related peptide therapies in selected tertiary headache centers [43].

The first follow-up after the initial consultation is crucial to evaluate treatment response and safety and, if necessary, change therapy and/or re-assess headache diagnosis. Ideally, the first follow-up after the first consultation or any treatment modification should be scheduled within a short interval (2–3 months) to evaluate treatment response [7]. However, this short timeframe might not always be feasible in clinical practice, and a first follow-up at 3 to 6 months is suggested. Conversely, when migraine symptoms are well-controlled and stable, follow-up visits should be tailored according to individual needs, rather than scheduled in advance. This position differs from other studies, where a programmed long-term follow-up is preferred [7, 13].

### Transition from primary care to specialist services

Although individuals with non-disabling and well-controlled disease do not generally require referral to specialized centers, more complex cases warrant escalation to higher levels of care. To guide referral decisions, the panel outlined clear criteria of eligibility for specialist consultation, including diagnosis of chronic migraine, overuse of acute treatments, inadequate response to traditional oral preventive therapies prescribed by the GP, and clinically relevant comorbidities.

Thus, individuals with chronic migraine, with or without overuse of acute medications, should also be directed to specialist evaluation due to the increased clinical complexity of their condition. In these cases, preventive therapies may reduce the risk or even resolve medication overuse [44], and their initiation should involve specialists to ensure adequate therapy and reduce reliance on acute treatments [9].

Beyond failure of traditional oral preventive therapies [9], individuals with relevant comorbidities should also be considered for specialist referral, particularly when such comorbidities exacerbate migraine symptoms and require a multidisciplinary approach [45].

Overall, a stepwise referral system in which the GP first directs migraine individuals to a territorial neurologist, who then decides whether referral to a tertiary headache center, is advisable. The panelists highlighted the key role of local regulations and care pathways in determining the feasibility of direct GP referrals to headache centers. Such referrals may be warranted when migraine management requires advanced therapies, available exclusively in headache centers, or in regions where territorial neurologist expertise or local service availability is insufficient.

### Optimizing care through a long-term collaboration between providers across levels of care

Integrated and dynamic management of individuals with migraine through a bi-directional collaboration between primary and specialist care is essential to establishing a flexible approach adaptable to their diverse needs. As such, communication and collaboration among GPs, territorial neurologists, and headache specialists should be optimized. In this context, the EHR was identified as the most appropriate tool to facilitate efficient communication between providers, although its usage remains heterogeneous across Italian regions [46]. This is particularly relevant for private visits, for which documentation cannot be automatically uploaded to the EHR; in those cases, medical records should be personally forwarded to GPs. The availability of a direct communication channel between headache centers and primary services would support GPs in their everyday activities, but efficient platforms are generally lacking. Instead, the role of telemedicine was particularly emphasized. As reported [47], telemedicine represents a valuable option, especially for follow-up visits, and should be implemented as it reduces both time commitments and healthcare costs.

AI tools are increasingly being explored in both primary and specialty care, with potential applications ranging from clinical decision support and information retrieval to administrative tasks [48, 49]. To date, most AI applications in primary care have involved deep learning for image analysis (e.g., diabetic retinopathy screening) and machine learning models based on EHR data, facilitating documentation screening, patient record analysis, and the identification of early intervention opportunities [49]. More recently, AI models have been developed to support gatekeeping processes, assisting in the triage and prioritization of referrals from primary to specialized care [50]. In the context of migraine care, AI-based models have demonstrated potential to improve diagnostic process, predict migraine attacks, and analyze neuroimaging and neurophysiological data, as well as to support treatment selection and predict treatment response, thereby enhancing headache management while reducing the administrative burden in clinical practice [51–53]. However, thus far, these applications have yet to be widely integrated into everyday clinical practice, as evidence regarding their clinical utility remains limited [53, 54].

## Limitations

Some limitations should be acknowledged. The restricted group of experts participating in the voting and the imbalance in the panel composition, characterized by a lower representation of GPs compared with specialists, represent the main limitation of this Delphi. The higher representation of specialists within the panelists reflects the selection of participants with documented expertise in migraine management. While specialists’ input is essential for defining evidence-based clinical pathways, this imbalance may have influenced the consensus, particularly regarding diagnostic approaches and therapeutic management in primary care. Moreover, the limited participation of GPs may have reduced the extent to which the consensus reflects real-world workload constraints and decision-making processes in general practice. Nevertheless, the inclusion of GPs, albeit limited, allowed the incorporation of primary care perspectives in migraine management, a field in which GP involvement in research remains limited, and should therefore also be considered as a distinct contribution of this work.

The lack of general neurologists among the panelists also represents a relevant limitation of this work. Their input could provide additional insights into the feasibility, organization, and practical implementation of optimal care strategies in routine clinical settings. Future initiatives should involve a greater representation of GPs, as well as general neurologists and other HCPs, to broaden perspectives and refine care pathways for migraine management across different levels of care.

In addition, the Delphi involved only Italian experts and panelists, limiting the generalizability of the results. The absence of representation from all Italian regions may have influenced the findings, considering the regional variability in healthcare regulations.

## Conclusions

This Delphi outlines an expert-based framework for optimizing migraine care, endorsing a shared, dynamic, and long-term management through appropriate operational interventions and structured collaboration across levels of care (Fig. 1). By translating internationally established best practices into a pragmatic care pathway including context-specific suggestions based on expert opinion, this consensus provides a realistic roadmap to improve migraine care across healthcare levels in Italy. This approach is intended to ensure adequate management of more complex migraine individuals while improving the economic sustainability of healthcare resources. Continuous educational initiatives aimed at enhancing GP awareness and knowledge of this disease, fostered through collaboration between scientific societies and healthcare institutions are essential. The involvement of supportive HCPs, ranging from nurses within general practice to non-neurological specialists and care managers in tertiary centers, could facilitate navigation across the care continuum, ensuring that individuals are directed to the most appropriate level of care and, when possible, redirected from tertiary centers back to primary care for long-term management.

The coordinated involvement of GPs, general neurologists, and headache specialists is necessary to optimize the migraine subject journey, relieve pressure on headache centers, and enable the provision of adequate care. Future projects are needed to ensure greater participation from GPs, to provide a clearer representation of their standpoint, strengthen their collaboration with specialists, and enhance the continuity of migraine care.

## Data Availability

N/A.
